# An Explainable Multi-Model Ensemble for Brain Hemorrhage Detection and Subtype Classification Using 34,366 NCCT Images

**DOI:** 10.3390/diagnostics16213514

**Published:** 2026-10-27

**Authors:** Seyyed Mohammad Mousavi, Hamid reza Samzadeh Kermani, Mahdi Mohammadi, Mohammad Mehdi Ghaemi

**Affiliations:** 1 Health Information Sciences Department, Faculty of Management and Medical Information Sciences, Kerman University of Medical Sciences, Kerman, Iran; m.mousavi@math.uk.ac.ir (S.M.M.); hhmmssvery@yahoo.com (H.r.S.K.); m.mohamadi989@yahoo.com (M.M.); 2 Medical Informatics Research Center, Institute for Futures Studies in Health, Kerman University of Medical Sciences, Kerman, Iran; 3 Health in Disasters and Emergencies Research Center, Institute for Futures Studies in Health, Kerman University of Medical Sciences, Kerman, Iran; 4 Modeling in Health Research Center, Institute for Futures Studies in Health, Kerman University of Medical Sciences, Kerman, Iran

**Keywords:** brain hemorrhage, deep learning, ensemble learning, non-contrast CT, grad-CAM, computer-aided diagnosis, medical image analysis

## Abstract

**Background:** Brain hemorrhage is a critical neurological emergency and it is essential to quickly and accurately identify the type of bleeding if treatment is to be properly guided and patient outcomes are to be improved. Even though non-contrast computed tomography (NCCT) is still the main imaging technique used for the initial evaluation, diagnostic difficulties continue due to overlapping radiological patterns and the limitations involved in manual interpretation. The purpose of this study was to create and validate an ensemble deep learning approach for the automated multi-class classification of brain hemorrhage subtypes based on NCCT images. **Methods**: Transfer learning was applied using three convolutional neural network architectures such as InceptionV3, Xception, and InceptionResNetV2. The models were trained independently and combined using an unweighted soft-voting strategy. The framework was evaluated on 34,366 NCCT images belonging to six diagnostic categories: EDH, SDH, SAH, IPH, IVH, and normal findings. **Results**: Xception achieved the highest performance among the individual models, whereas the ensemble produced the best overall results. Using the final test-set results, the ensemble achieved an accuracy of 95.35%, with macro-averaged precision, recall, and F1-score of approximately 95%. Grad-CAM visualizations showed that the model frequently attended to anatomically plausible regions associated with the predicted hemorrhage categories. **Conclusions**: The findings suggest that combining complementary CNN architectures may improve internal test-set performance for six-class NCCT image classification. However, because the study used an image-level split from a single public dataset without external validation, the proposed framework should be considered a research-stage decision-support approach rather than a clinically validated diagnostic system.

## 1. Introduction

Brain hemorrhage is a life-threatening neurological emergency in which rapid diagnosis and accurate identification of the hemorrhage subtype are essential for appropriate treatment and improved patient outcomes [1,2,3]. Based on its anatomical location, ICH is commonly classified into epidural hematoma (EDH), subdural hematoma (SDH), subarachnoid hemorrhage (SAH), intraparenchymal hemorrhage (IPH), and intraventricular hemorrhage (IVH). These subtypes differ in their anatomical distribution, radiological appearance, clinical consequences, and management requirements. However, their imaging manifestations may overlap, particularly in emergency settings and in cases with small, atypical, or evolving hemorrhagic lesions.

Non-contrast computed tomography (NCCT) remains the primary imaging modality for the initial assessment of suspected intracranial hemorrhage because it is rapid, widely available, and highly sensitive to acute blood products [4,5]. Nevertheless, interpretation of NCCT examinations depends heavily on the experience and workload of the radiologist. Subtle hemorrhagic findings, a large number of axial slices, and the time-sensitive nature of emergency diagnosis may increase the risk of delayed or inaccurate interpretation. These challenges have motivated the development of computer-aided diagnostic systems that can support radiologists by providing rapid and consistent image analysis.

Recent advances in deep learning have enabled the automatic extraction of discriminative representations directly from medical images. Convolutional neural networks, transfer-learning models, and ensemble-learning methods have demonstrated considerable potential for detecting brain hemorrhage and distinguishing among hemorrhage subtypes [6]. However, existing studies vary substantially in terms of the clinical task, number of diagnostic classes, dataset size, image-level or patient-level evaluation, model architecture, and validation strategy. Consequently, performance values reported across studies cannot always be compared directly. A detailed analysis of previous work and its methodological characteristics is presented in Section 2.

Despite the progress achieved in this field, several challenges remain. Many previous investigations have focused primarily on binary hemorrhage detection, whereas comprehensive classification of multiple hemorrhage subtypes is more clinically demanding. In addition, several studies have relied on a single deep learning architecture, limited datasets, or internal validation without external testing. The interpretability of ensemble models has also received comparatively limited attention, although clinically meaningful explanations are important for the adoption of artificial intelligence in radiology.

Therefore, this study proposes an explainable multi-model ensemble framework for six-class classification of NCCT images, including EDH, SDH, SAH, IPH, IVH, and normal cases. The framework combines InceptionV3, Xception, and InceptionResNetV2 through a voting-based strategy and uses Grad-CAM to visualize the image regions associated with the model predictions. The main objectives of this study were to: (i) evaluate the performance of three transfer-learning CNN architectures for multi-class hemorrhage classification; (ii) investigate whether their combination improves classification performance; and (iii) assess whether the ensemble predictions are associated with anatomically and radiologically relevant regions.

## 2. Related Works

Deep learning has been widely investigated for the automated analysis of head CT images. Several studies have evaluated individual CNN architectures for the detection or classification of intracranial hemorrhage. Grewal et al. [7] developed RADnet, a recurrent attention-based DenseNet that incorporated information from neighboring CT slices to provide additional three-dimensional context for hemorrhage detection. Ammar et al. [8] compared several CNN architectures for multi-class intracranial hemorrhage classification and reported the highest overall accuracy using VGG16. Other studies have investigated Xception-based architectures and reported promising results, particularly when feature-fusion strategies or double-branch designs were incorporated [9,10].

Inception-family architectures have also been examined for brain hemorrhage analysis. Hassan and Aljobouri [11] evaluated InceptionResNetV2 together with DenseNet201 for the classification of selected hemorrhage subtypes. These studies suggest that transfer-learning architectures can provide strong performance when sufficient preprocessing and optimization are applied. However, the reported results are influenced by differences in dataset composition, class definitions, preprocessing protocols, and evaluation procedures.

Because different CNN architectures may learn complementary visual representations, ensemble methods have been proposed to improve the robustness of brain hemorrhage classification. Hussain et al. [12] developed an attention-based ResNet framework that combined dimensionality reduction and synthetic data generation to address feature complexity and class imbalance. Other investigations have combined VGG16, ResNet50, and DenseNet121 using voting, feature fusion, or recurrent components [13,14]. Kuo et al. [15] also evaluated a cross-architecture system involving EfficientNet-B4, Xception, and InceptionV3 for the detection of acute intracranial hemorrhage.

Although these studies demonstrate the potential of combining multiple models, several limitations remain. Some investigations focus on binary detection rather than multi-class subtype classification, while others use different numbers of classes or evaluate only selected hemorrhage types. Moreover, direct comparisons are difficult because the datasets, partitioning strategies, and performance metrics are not consistent across studies. In addition, the interpretability of ensemble predictions is not always systematically evaluated.

Data augmentation has commonly been used to increase the effective size of medical imaging datasets and reduce overfitting. Rotation, translation, scaling, flipping, and zooming have been evaluated as strategies for improving model generalization [16]. More recent approaches have investigated anatomy-aware augmentation methods that attempt to preserve clinically meaningful spatial relationships [17]. Attention mechanisms and multi-scale feature extraction have also been used to improve sensitivity to hemorrhagic regions and to support efficient analysis in emergency workflows [18]. Large-scale studies training 3D convolutional neural networks on 35,000 head CT examinations achieved 99.0% area under the ROC curve for hemorrhage detection [19]. Hierarchical classification frameworks that first distinguish hemorrhagic from non-hemorrhagic cases before performing fine-grained subtype classification achieved 93.1% accuracy for subtype classification on datasets of 20,000 NCCT images [20].

Beyond image-only classification, radiomics and multimodal approaches have incorporated quantitative image features together with clinical variables. For example, the integration of radiomics features with information such as patient age and hemorrhage location has been investigated for differentiating etiologies of spontaneous intracranial hemorrhage [21]. These findings indicate that clinical metadata may provide complementary information, particularly for radiologically heterogeneous categories such as IPH.

The reviewed literature indicates that several important challenges remain unresolved. First, a substantial proportion of previous studies have focused on binary hemorrhage detection rather than simultaneous classification of all major hemorrhage subtypes and normal cases. Second, studies often evaluate a single architecture or use datasets and protocols that limit direct comparison across methods. Third, external validation is uncommon, and the generalizability of reported performance across institutions and imaging protocols remains uncertain. Finally, although explainability is important for clinical acceptance, relatively few ensemble-based studies have systematically examined whether model attention corresponds to anatomically relevant hemorrhagic regions.

The three architectures were selected because they provide complementary feature representations: InceptionV3 captures multiscale patterns through factorized convolutions, Xception efficiently extracts fine-grained spatial features using depthwise separable convolutions, and InceptionResNetV2 combines multiscale Inception modules with residual connections. Their use is also supported by previous studies reporting promising results for intracranial hemorrhage classification [8,9,10,11].

The present study addresses these issues by evaluating three complementary transfer-learning architectures such as InceptionV3, Xception, and InceptionResNetV2 within a soft voting-based ensemble framework. The proposed system performs six-class classification using 34,366 NCCT images and incorporates Grad-CAM visualization to examine the anatomical relevance of the model predictions. A comparison of representative previous studies is provided in Table 1.

## 3. Materials and Methods

This study developed and evaluated an explainable deep-learning framework for six-class classification of NCCT brain images. The diagnostic categories were epidural hematoma, subdural hematoma, subarachnoid hemorrhage, intraparenchymal hemorrhage, intraventricular hemorrhage, and normal findings. The methodological workflow consisted of six main stages: dataset selection and class balancing, image preprocessing, stratified dataset partitioning, model training using transfer learning, construction of a soft-voting ensemble, and quantitative and explainability-based evaluation.

First, the publicly available NCCT dataset was curated by retaining all available EDH images and randomly selecting images from the other diagnostic categories to reduce the original class imbalance. The images were then standardized through resizing, intensity normalization, conversion from grayscale to three channels, and noise reduction. The resulting dataset was divided into training, validation, and independent test subsets using stratified random sampling with a fixed random seed. Data augmentation was applied only to the training subset.

**Figure 1:**
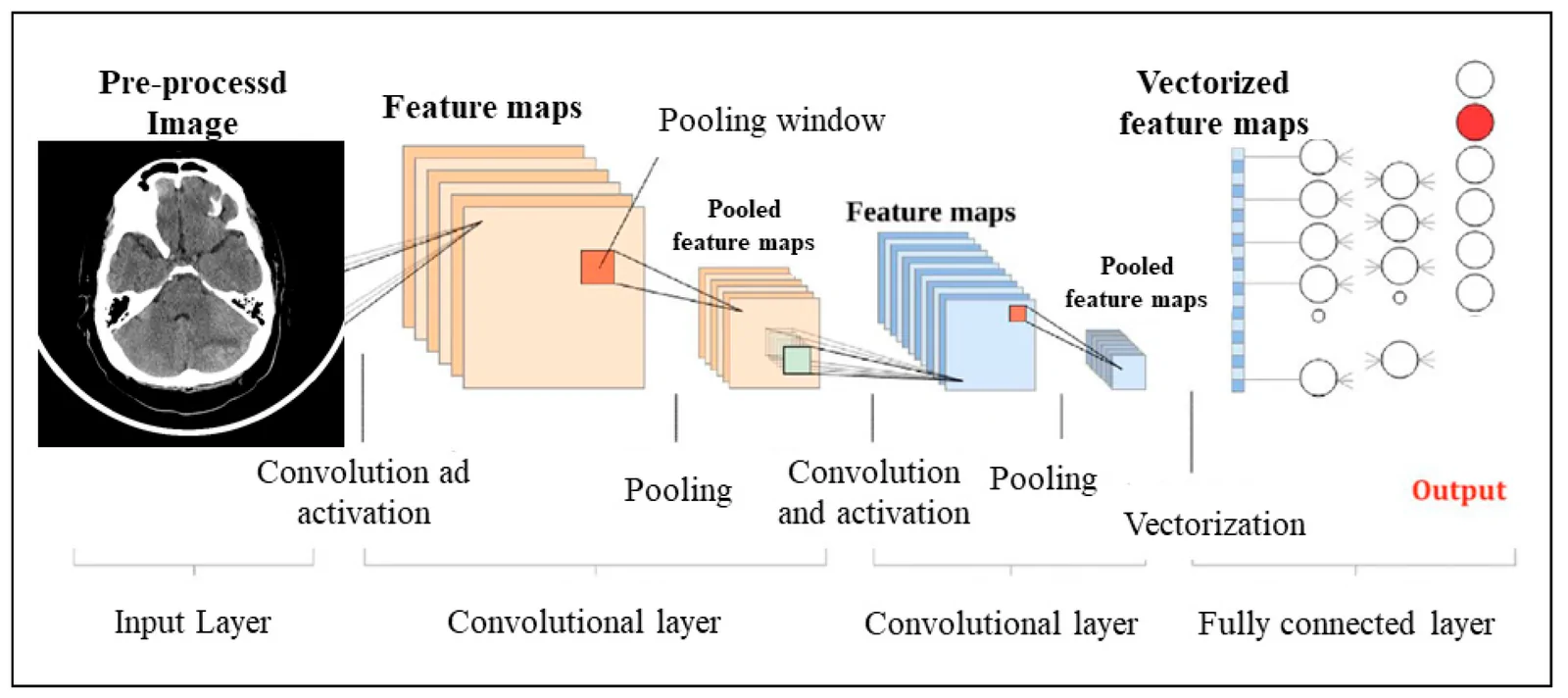
Research Methodology Framework.

Three ImageNet-pretrained convolutional neural networks such as InceptionV3, Xception, and InceptionResNetV2, were independently fine-tuned for six-class classification. Their predicted class probabilities were subsequently combined using a soft-voting strategy to obtain the final ensemble prediction. Model performance was assessed on the independent test set using accuracy, precision, recall, F1-score, AUC, and 95% confidence intervals where applicable. Grad-CAM was additionally applied to selected test images to visualize the image regions contributing to the model predictions. Figure 1 summarizes the complete methodological workflow.

### 3.1. Dataset and Preprocessing

We used the publicly available Brain Hemorrhage Multiclass dataset (https://www.kaggle.com/datasets/yogeshkn/brain-hemorrhage-multiclass, accessed on 10 August 2026), which contains de-identified non-contrast head CT images organized into six diagnostic categories: EDH, SDH, SAH, IPH, IVH, and normal findings. In the present study, the individual CT slices were treated as the unit of analysis. Because patient-level identifiers and complete examination-level information were not available in the public dataset, the study was designed as an image-level classification analysis rather than a patient-level diagnostic evaluation.

The original dataset showed substantial variation in the number of images across diagnostic categories. Specifically, it included 4366 images of EDH, 8100 normal images, 9540 images of IPH, 8148 images of IVH, 8040 images of SAH, and 8880 images of SDH. Thus, EDH represented the least frequent category, whereas IPH constituted the largest category in the original dataset.

**Figure 2:**
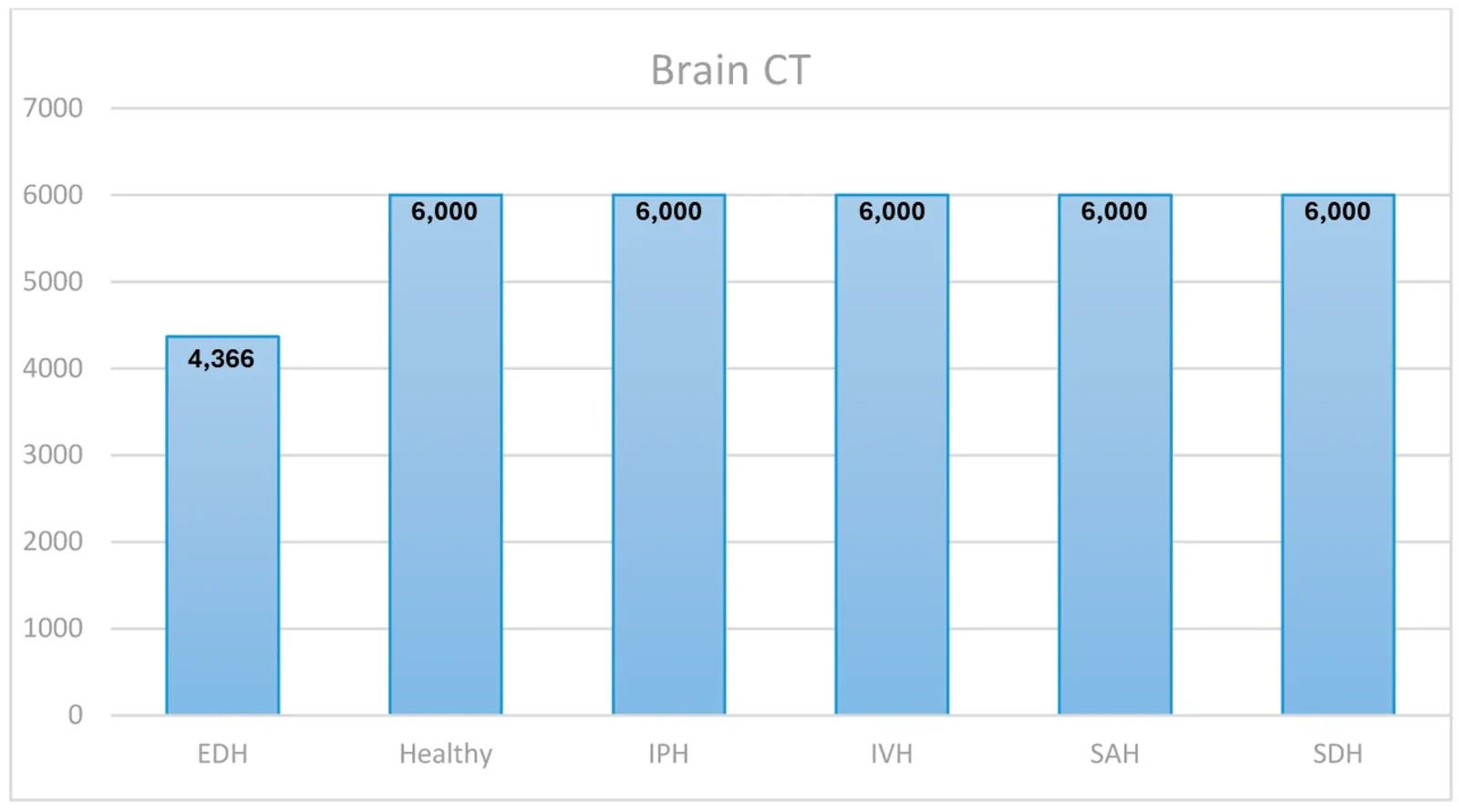
Dataset representation.

**Figure 3:**
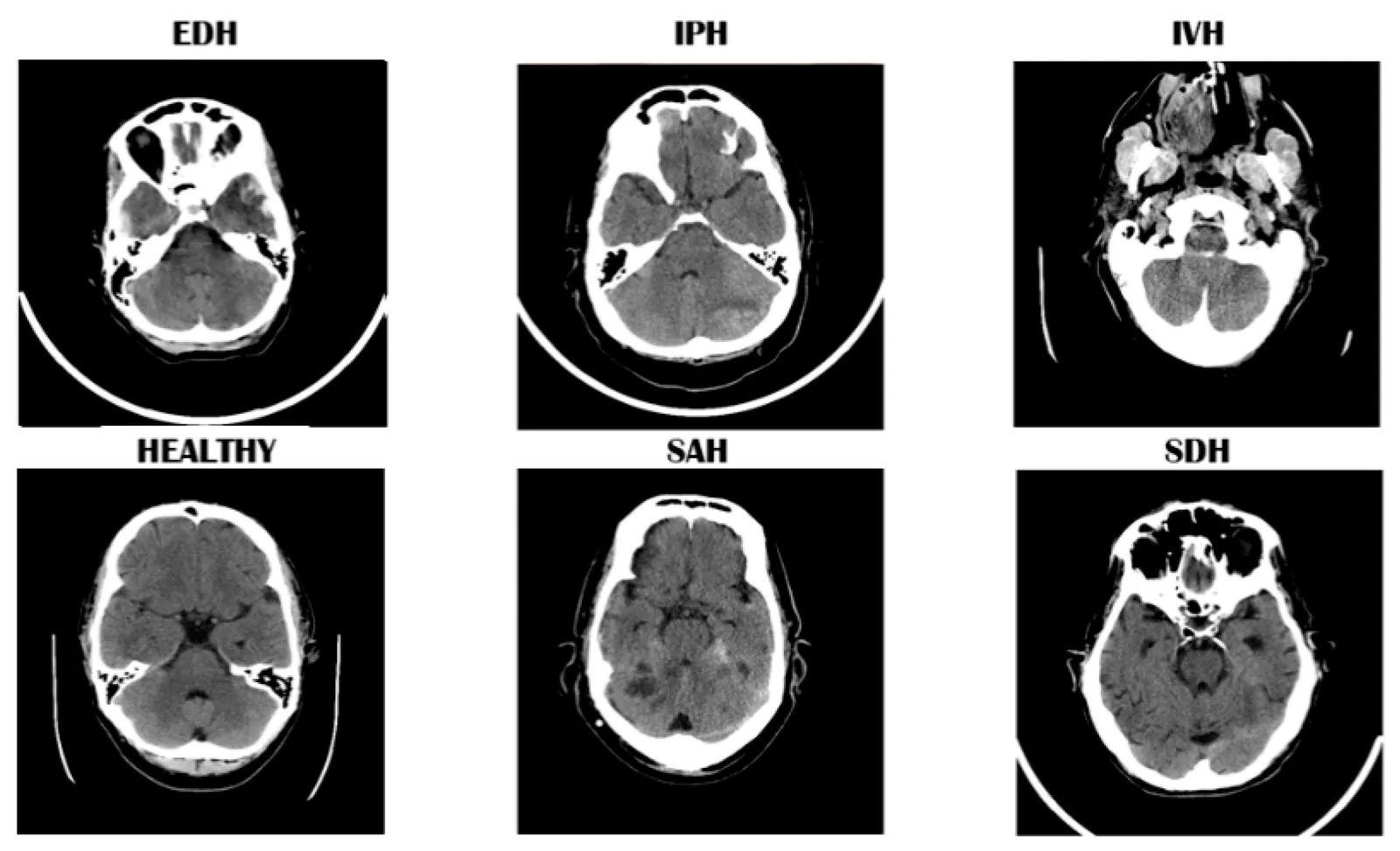
Visual representation of Dataset.

To mitigate the potential influence of class imbalance during model development, all available EDH images were retained, while a random subset of 6000 images was selected from each of the remaining diagnostic categories. This sampling strategy resulted in a final dataset of 34,366 NCCT images, comprising 4366 EDH images and 6000 images for each of the healthy, IPH, IVH, SAH, and SDH categories. Rather than achieving complete class equivalence, this approach reduced the disproportionate contribution of the larger classes while preserving all observations from the minority EDH class. The distributions of the original and final datasets are illustrated in Figure 2, and representative images from each class are presented in Figure 3.

Before model training, all images underwent the same preprocessing pipeline. Each grayscale NCCT image was resized to 299 × 299 pixels, corresponding to the input size of the selected CNN architectures. Pixel intensities were normalized using min–max scaling. Because the pretrained networks require three-channel input, the single grayscale channel was replicated across the red, green, and blue channels. Gaussian filtering was subsequently applied to reduce high-frequency noise and acquisition-related artifacts. The same deterministic preprocessing procedure was applied to the training, validation, and test images, whereas stochastic augmentation was restricted to the training subset.

### 3.2. Data Augmentation

In order to improve the model’s ability to generalize and reduce the problem of overfitting, data augmentation was carried out on the training set using the Keras ImageDataGenerator [22]. This technique consists of applying random transformations to the images at each epoch so as to make the model good against changes in the position, scale, and orientation of the images. The parameters used for augmentation were carefully chosen so as to introduce variations that are plausible from a physiological point of view while at the same time preserving anatomical integrity:

- Random rotation up to 30 degrees
- Horizontal and vertical shifts up to 10%
- Shear distortion
- Zoom range up to 20%
- Horizontal flipping

The augmentation parameters were chosen because earlier studies had shown that moderate geometric transformations greatly enhance the model’s ability to generalize when it comes to detecting hemorrhage from CT scans without causing unrealistic artifacts [16,17].

### 3.3. Model Architectures

The study used transfer learning involving pre-trained CNNs for the purpose of feature extraction and classification. By using transfer learning, knowledge gained from large-scale datasets such as ImageNet is applied to the target domain when only a limited amount of labeled data is available, which in turn improves convergence and generalization. The following three complementary architectures were implemented:

#### 3.3.1. InceptionV3

The architecture makes use of multi-scale factorized convolutions in order to capture hierarchical features over a range of receptive fields [7,8]; its design includes parallel convolutional operations with different kernel sizes, which allows for the simultaneous detection of both fine-grained and coarse features, the latter of which are especially important for distinguishing between hemorrhage subtypes that have different spatial extents. This multiscale design is suitable for NCCT analysis because hemorrhagic lesions vary in size, shape, and anatomical distribution. InceptionV3 has also shown promising performance in previous multi-class intracranial hemorrhage studies [8].

#### 3.3.2. Xception

The model uses depthwise separable convolutions in order to efficiently extract fine-grained spatial features [9,10]. Since the spatial and channel-wise correlations are decoupled, Xception is able to achieve improved representational efficiency without compromising computational tractability, which is why it is especially effective at capturing the boundary features that distinguish one type of hemorrhage from another. This design may be advantageous for detecting fine-grained patterns, lesion boundaries, and subtle density differences in NCCT images. Previous Xception-based studies have also reported strong performance in hemorrhage detection and subtype classification [9,10].

#### 3.3.3. InceptionResNetV2

By combining Inception modules with residual connections, this hybrid architecture allows for deeper feature learning together with better gradient flow, as demonstrated by Hassan and Aljobouri [11] in their InceptionResNetV2-based model for intracranial hemorrhage detection. The use of skip connections helps in the training of deeper networks by overcoming the vanishing gradient problem and thus makes it possible to extract more abstract feature representations. The combination of multiscale Inception modules and residual connections enables the model to learn hierarchical representations while facilitating gradient propagation. This architecture was selected as a complementary ensemble component and has previously been investigated for CT-based intracranial hemorrhage classification [11].

All the models had the same architecture for the classification head. The feature maps were reduced by means of GlobalAveragePooling2D and then converted into one-dimensional vectors. For improved generalizability and as a form of regularization, dense layers with ReLU activation and dropout rates of 0.3 were used. A SoftMax output layer containing six neurons was employed to give the probability distributions for the various diagnostic categories.

### 3.4. Dataset Partitioning

After class-specific sampling, the final dataset of 34,366 CT slices was divided into training, validation, and test sets using stratified random sampling with a fixed random seed of 42. The split ratio was 85% for training, 10% for validation, and 5% for testing. Stratification was applied to preserve the class distribution across the three subsets.

Importantly, the split was performed at the image level because the publicly available dataset did not contain PatientID, ExaminationID, accession number, study identifier, or any other metadata that could link individual slices to the same patient or CT examination. Therefore, a patient-level or examination-level split before image sampling was not possible. The test set should consequently be regarded as an internal image-level test set rather than an independent patient-level or examination-level test set. Class-specific sampling was performed only on the training subset to avoid modifying the class distribution in the validation and test sets, which were kept representative of the original dataset.

### 3.5. Ensemble Strategy

An ensemble framework was created by combining the predictions from the three different architectures through a voting approach. The models were selected not only according to their individual accuracy but also according to their potential to produce complementary predictions. Therefore, InceptionResNetV2 was retained despite its lower standalone performance, as its residual-based representations may provide prediction patterns that differ from those of InceptionV3 and Xception.

### 3.6. Training Protocol

All three architectures were initialized with ImageNet-pretrained weights. For transfer learning, the convolutional base of each model was initially frozen, and only the classification head (global average pooling, dense layers with ReLU activation, dropout 0.3, and final SoftMax layer) was trained for the first 5 epochs. Subsequently, the entire network was unfrozen and fine-tuned end-to-end for up to 30 epochs. The Adam optimizer was used with an initial learning rate of 0.03, selected via grid during hyperparameter tuning. Although this learning rate is higher than commonly reported values for Adam, it was chosen because it provided faster initial convergence without instability in our setting, in combination with learning-rate scheduling and early stopping. Learning-rate scheduling was implemented using ReduceLROnPlateau with a patience of 3 epochs, a reduction factor of 0.1, and a minimum learning rate of 1 × 10^−6^. Early stopping was applied with a patience of 9 epochs, monitoring the validation loss, and restoring the best weights. The batch size was set to 32, and the loss function was sparse categorical cross-entropy. All hyperparameters are presented in Table 2.

All images were preprocessed as described in Section 3.1. Data augmentation was applied only to the training set using random rotation (±30°), horizontal and vertical shifts (up to 10%), shear, zoom (up to 20%), and horizontal flipping.

**Table 2:** Hyperparameters of the proposed model.

Hyperparameters	
Activation Function	ReLU
Epochs	30
Batch Size	32
Optimizer	Adam
Loss Function	sparse_categorical_crossentropy
Dropout Rate	0.3
Learning Rate Schedule	ReduceLROnPlateau (patience = 3)
Early Stopping Patience	9 Epochs
Initial Learning Rate	0.03

### 3.7. Performance Evaluation

After the training had been carried out, the generalization performance of the proposed models was assessed by means of the independent test dataset. The models’ performance was measured using a standard collection of classification metrics, namely accuracy, precision, recall, and the F1-Score. In order to increase the transparency and clinical trustworthiness of the ensemble model, Grad-CAM was used to visualize the areas of interest that had contributed most to the model’s classification decisions. Grad-CAM produces coarse localization maps which highlight the important areas in the input image for predicting a particular class by making use of the gradients of the target class flowing into the final convolutional layer. This method allows clinicians to check whether the model’s area of focus corresponds with the radiologically relevant hemorrhagic regions, thus providing the kind of interpretability that is essential for clinical adoption. Table 3 gives the evaluation metrics and their mathematical formulations.

## 4. Results

The performance of the proposed models when tested on the CT datasets is systematically assessed by means of a wide range of quantitative and analytical measures, including overall accuracy, performance metrics broken down by class, an analysis of the confusion matrix, and an examination of the training dynamics.

### 4.1. Model Training Progress

**Figure 4:**
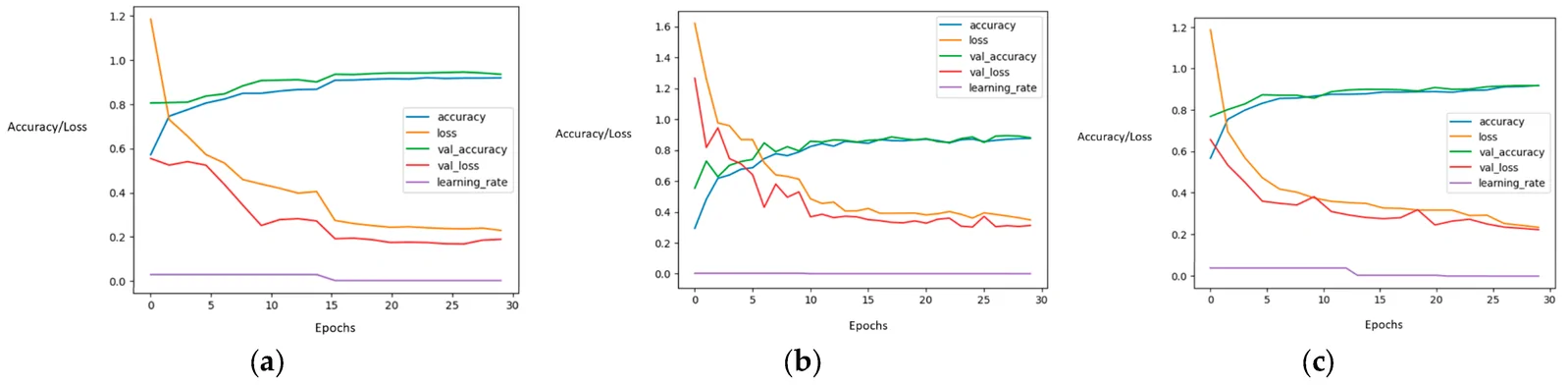
Model training and validation curves. (**a**) Xception. (**b**) Inception-ResNetV2. (**c**) InceptionV3.

The training dynamics of the evaluated architectures were analyzed through epoch-wise trajectories of accuracy and loss, as depicted in Figure 4. These curves provide insight into convergence behavior, optimization efficiency, and generalization characteristics of each model when applied to NCCT-based brain hemorrhage classification.

The training and validation show distinct convergence patterns across the three architectures. Xception exhibited the most rapid improvement, with accuracy increasing sharply during the initial epochs before stabilizing at a relatively high level. Its training and validation accuracy curves remained broadly aligned, while both loss curves declined, indicating stable optimization over the course of training. InceptionV3 showed a more gradual but generally consistent learning pattern, accuracy improved over successive epochs, training and validation performance approached similar levels toward the end of training as the losses decreased. InceptionResNetV2 displayed slower convergence and greater epoch-to-epoch variability, particularly in its validation curves. Despite these fluctuations, its overall accuracy increased and its loss decreased, suggesting continued learning rather than persistent deterioration in validation performance. Across the three models, the later curves show smaller changes than those in the early epochs, consistent with a trend toward convergence. The absence of sustained divergence between training and validation accuracy provides no clear evidence of pronounced overfitting in these curves, although the greater validation variability for InceptionResNetV2 indicates less stable training behavior.

### 4.2. Individual Model Performance

The quantitative results obtained with the three convolutional neural network architectures, each of which was trained independently, on the NCCT test set are given in Table 4. The evaluation was carried out by means of the usual classification metrics in order to thoroughly assess both the overall predictive performance and the ability to discriminate between classes.

**Figure 5:**
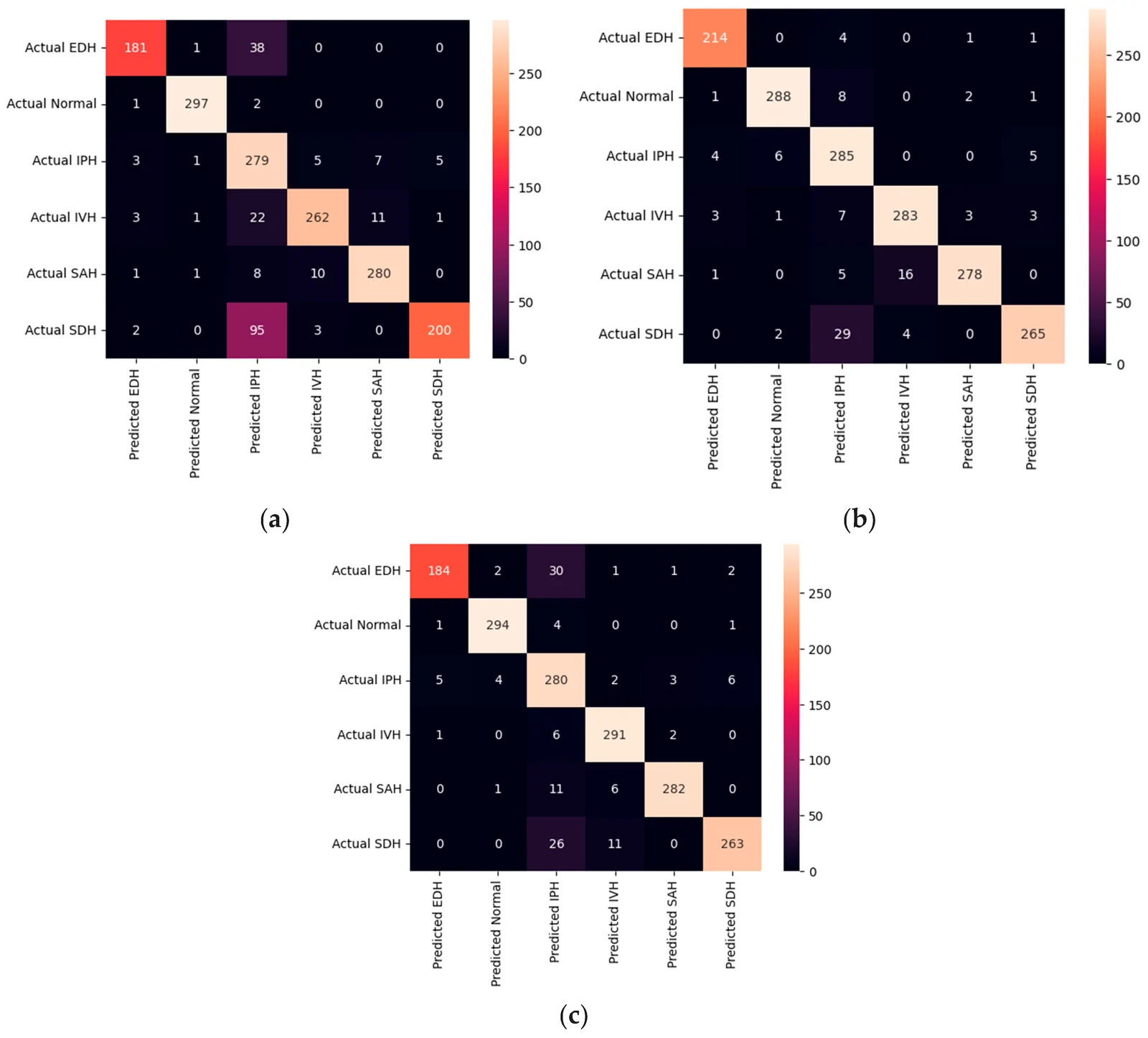
Confusion matrix of the CT model. (**a**) InceptionResnetV2. (**b**) Xception network. (**c**) InceptionV3 network.

Of the individual models, Xception obtained the best overall results, achieving an accuracy of 93%, together with consistently high precision, recall, and F1-score values of 94% and an AUC of 0.9956. This balance indicates that the model not only made mostly correct predictions but also maintained a comparable trade-off between false positives and false negatives, a result probably attributable to its efficient depthwise separable convolutional architecture for extracting discriminative spatial features. InceptionV3 achieved a closely comparable accuracy of 92% with precision, recall, and F1-score values of 93%, and in fact attained the highest AUC among the individual models, indicating strong discrimination across the six diagnostic categories despite its marginally lower overall accuracy. In contrast, InceptionResNetV2 showed considerably poorer performance, with an accuracy of 87%, precision of 90%, recall of 87%, an F1-score of 87%, and an AUC of 0.9893, suggesting reduced generalization and a greater likelihood of false negatives relative to the other two architectures. Overall, the confusion matrices in Figure 5 and the class-wise results in Table 4 further confirm that Xception and InceptionV3 exhibit superior and more stable classification behavior than InceptionResNetV2.

The class-wise analysis in Table 5 reveals consistently strong performance across most categories, with some notable class-specific differences. The healthy class was identified almost perfectly by all three models, with F1-scores of 96% to 99%, indicating that the networks rarely misclassified normal scans as hemorrhagic. SAH was also reliably classified by all architectures, with F1-scores of 94% to 95%; its relatively distinct radiological appearance, characterized by hyperdense material within the sulci and basal cisterns, likely accounts for this consistent identification [12].

All the individual models showed poorer classification performance for intraparenchymal hemorrhage, with F1-scores ranging from 75% to 89%. This decreased performance probably results from the radiological heterogeneity of IPH, since its appearance on NCCT varies greatly depending on the stage of the hemorrhage, the presence of edema, and its anatomical location; in some instances, especially during the hyperacute phase, it can even have the same density as normal brain parenchyma. This temporal and visual variability leads to intrinsic ambiguity and makes IPH more difficult to classify reliably than more morphologically distinct hemorrhage types. The class-wise results also reveal complementary error patterns: InceptionResNetV2 achieved an IPH precision of only 63%, indicating a marked tendency to over-call this class, whereas InceptionV3 showed the opposite behavior, and Xception provided the best IPH balance. Additional class-specific weaknesses were observed for InceptionResNetV2 in SDH and for InceptionV3 in EDH, suggesting that misclassifications were concentrated in a small number of visually confusable subtype pairs rather than being distributed uniformly across classes.

**Figure 6:**
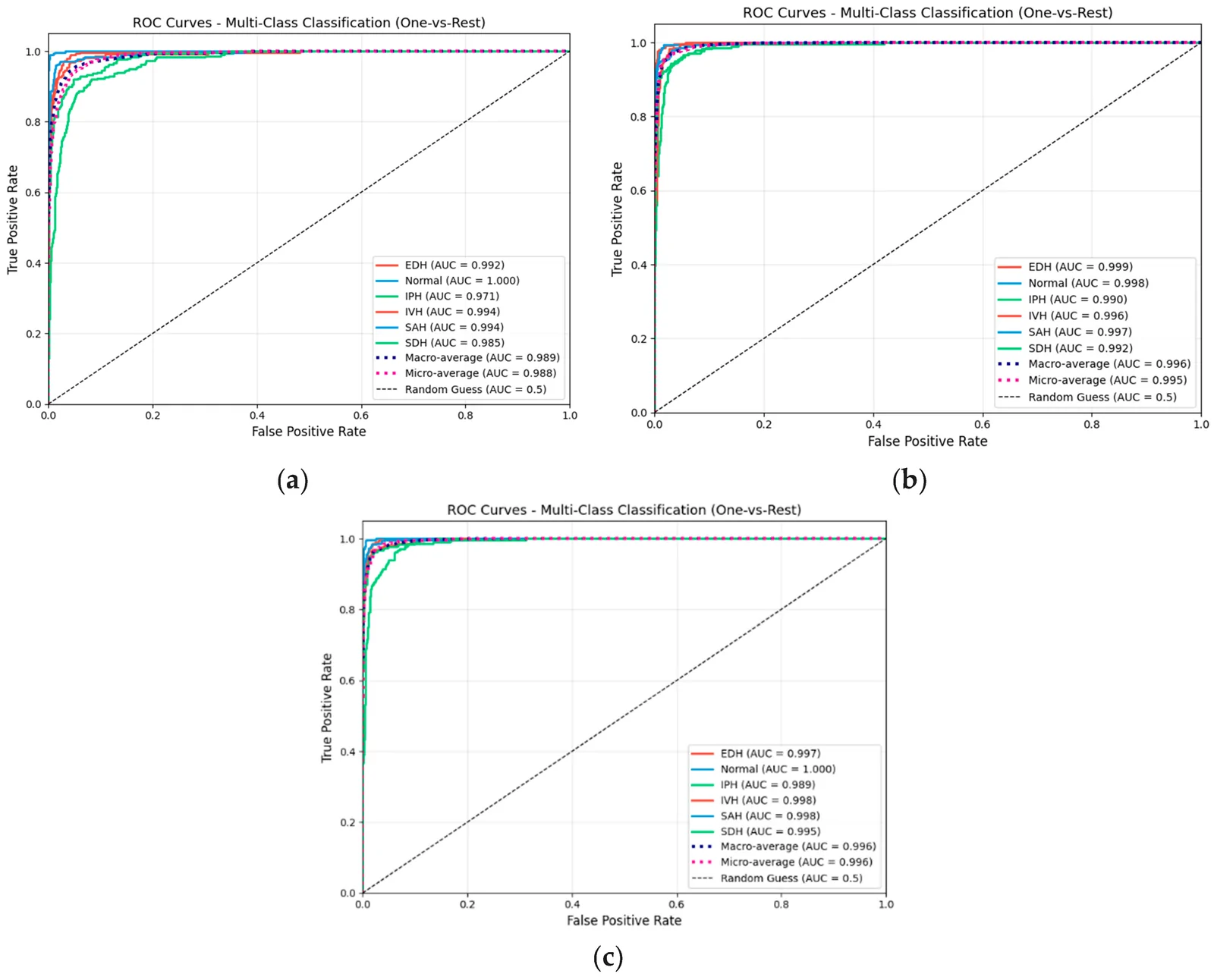
ROC of the CT model. (**a**) InceptionResnetV2, (**b**) Xception, (**c**) InceptionV3.

Figure 6 presents the ROC curves for the evaluated CT-based models across the six diagnostic categories. All models demonstrated excellent discriminative ability, with high AUC values: 0.9961 for InceptionV3, 0.9956 for Xception, and 0.9893 for InceptionResNetV2. InceptionV3 achieved the highest overall AUC among single models, indicating marginally superior class-separation capability across decision thresholds, whereas Xception showed a closely comparable performance. Although InceptionResNetV2 yielded a lower AUC than the other two architectures, its ROC curve still indicated strong discrimination. The class-wise ROC analysis further demonstrated consistently high AUC values across the hemorrhage subtypes and normal cases, with the lowest discrimination generally observed for IPH, reflecting its greater radiological heterogeneity. The ROC findings support the strong discriminative performance of the evaluated architectures; however, they should be interpreted in conjunction with external validation results rather than as evidence of clinical readiness alone.

### 4.3. Ensemble Model Performance

To improve classification robustness and reduce the limitations of the individual models, the predictions generated by InceptionV3, Xception, and InceptionResNetV2 were combined using a soft-voting ensemble strategy. The ensemble achieved an overall accuracy of 95.34%, with precision, recall, and F1-score values of approximately 95%. These results indicate a balanced classification performance across the six diagnostic categories.

The class-wise results presented in Table 6 demonstrate strong performance across all categories. The ensemble achieved F1-scores of 97% for EDH, 97% for normal cases, 91% for IPH, 96% for IVH, 97% for SAH, and 95% for SDH. The corresponding AUC values ranged from 0.9933 to 0.9993, confirming discriminative performance across the evaluated classes. Although IPH remained the most challenging category, the ensemble achieved an F1-score of 91%, which was higher than the corresponding values obtained by the individual models.

**Figure 7:**
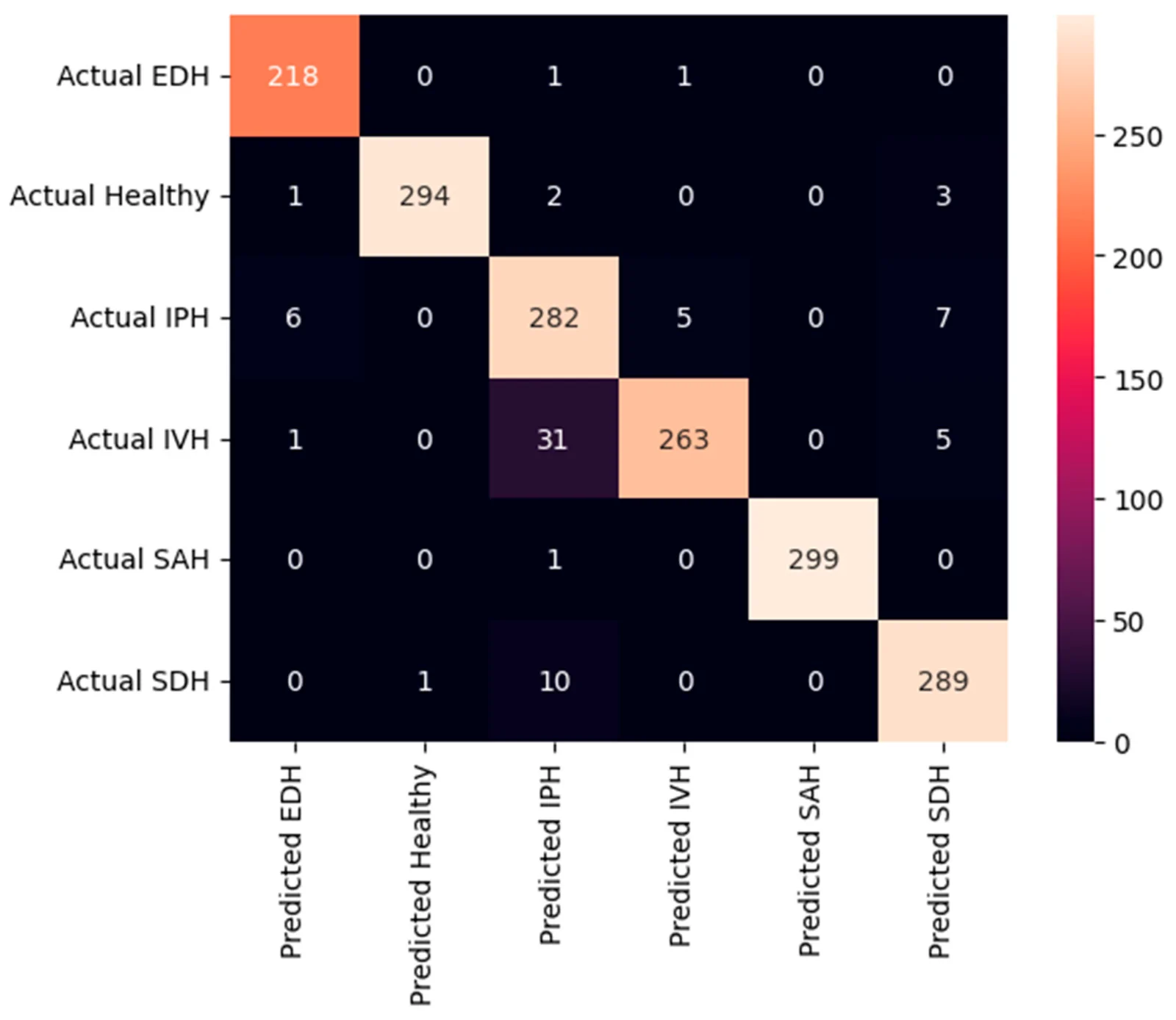
Dataset confusion matrix for Ensemble Model.

The confusion matrix of the ensemble model is shown in Figure 7. The matrix demonstrates that most samples were correctly classified across all six categories, with relatively few misclassifications. The highest classification consistency was observed for the normal, SAH, and IVH categories. The remaining errors were mainly associated with the visually overlapping features of hemorrhagic subtypes, particularly IPH and SDH. Overall, the confusion matrix supports the high accuracy and balanced class-wise performance reported in Table 6.

**Figure 8:**
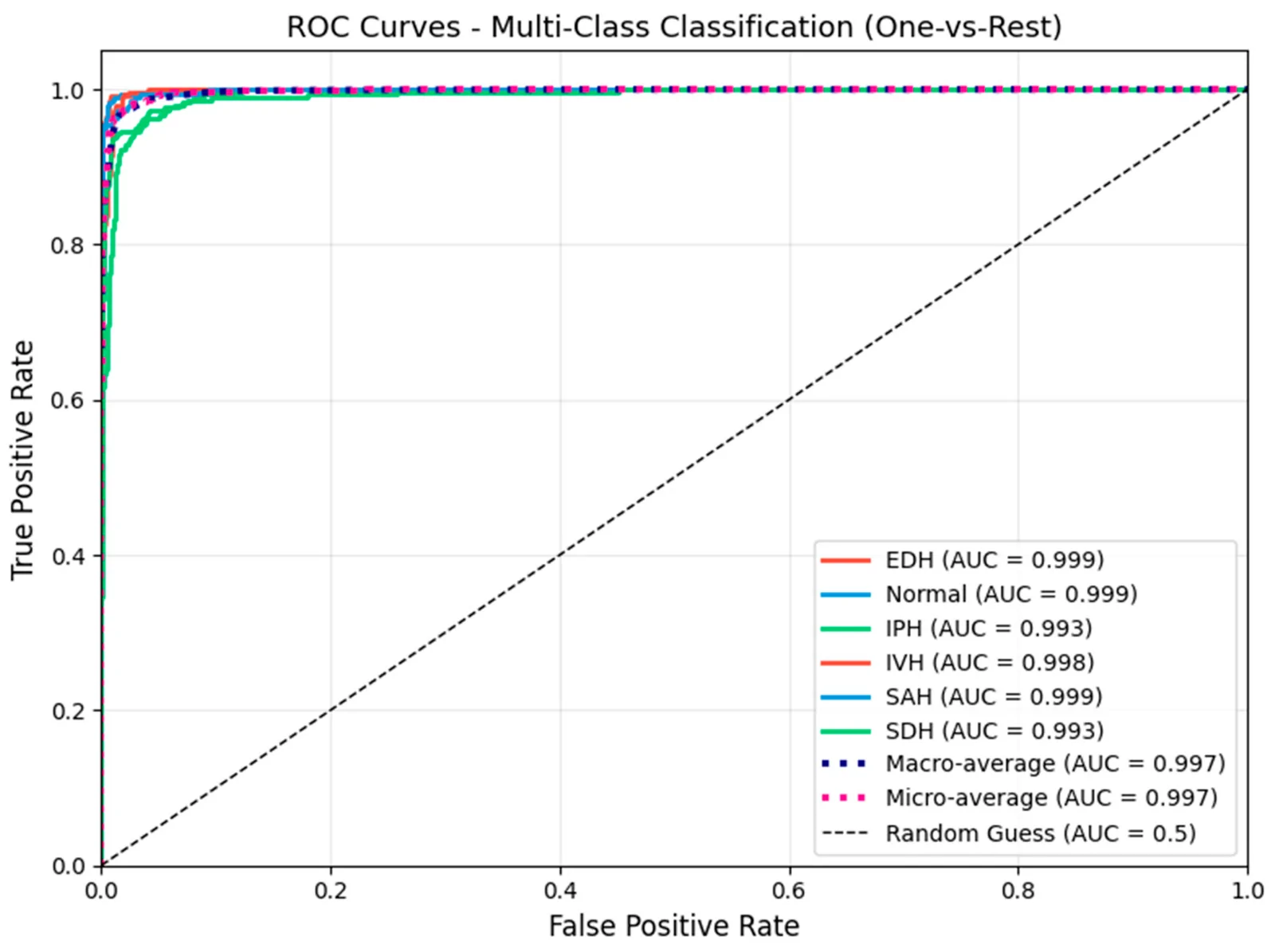
ROC of the Ensemble model.

The ROC curves of the ensemble model are presented in Figure 8. The model showed excellent discrimination across all six classes, with class-specific AUC values ranging from 0.9933 to 0.9993. The highest AUC was observed for EDH, whereas IPH and SDH showed slightly lower values, consistent with their greater radiological heterogeneity and potential overlap with other hemorrhage patterns. Nevertheless, the high AUC values indicate that the ensemble maintained strong class-separation ability across a broad range of decision thresholds.

The improved performance of the ensemble can be attributed to the complementary representations learned by the three architectures. Xception contributes fine-grained spatial features through depthwise separable convolutions, InceptionV3 captures multiscale patterns through factorized convolutional branches, and InceptionResNetV2 provides residual multiscale representations. Although InceptionResNetV2 showed lower standalone performance, its inclusion may have contributed complementary prediction patterns that helped reduce architecture-specific errors. Although a more exhaustive ablation was not performed, the comparison between each individual architecture and the full ensemble provides initial evidence that combining complementary models improves slice-level classification performance relative to any single architecture alone.

Compared with the individual models, the ensemble achieved a higher overall accuracy than Xception, InceptionV3, and InceptionResNetV2, whose accuracies were 93.779%, 92.674%, and 87.151%, respectively. These findings indicate that combining multiple CNN architectures can improve the reliability of six-class NCCT classification. Nevertheless, the reported results represent internal evaluation on the available test set; therefore, external validation using larger, multi-institutional datasets is required before the model can be considered for clinical implementation.

### 4.4. Model Explainability and Attention Visualization

**Figure 9:**
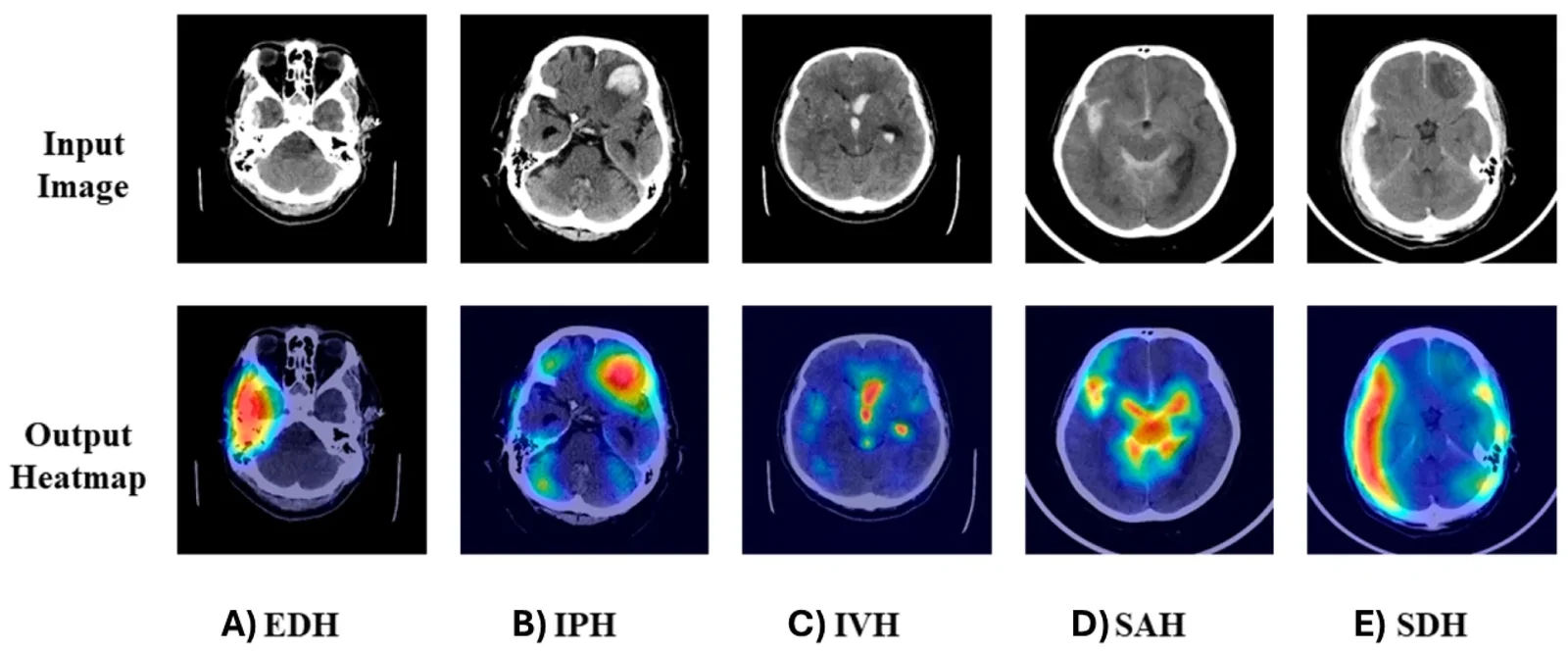
Visualizations demonstrating model attention regions for classified cases across all hemorrhage classes. For each class: (**top image**) Original NCCT image, (**bottom image**) Grad-CAM heatmap overlay. (**A**) Epidural Hematoma (EDH), (**B**) Intraparenchymal Hemorrhage (IPH), (**C**) Intraventricular Hemorrhage (IVH), (**D**) Subarachnoid Hemorrhage (SAH), (**E**) Subdural Hematoma (SDH) case.

Figure 9 presents the Grad-CAM visualizations for selected cases from each hemorrhage subtype. The heatmaps show that the ensemble model correctly focuses on important areas matching the typical anatomical locations for each type of hemorrhage.

As shown in Figure 9, the model’s attention maps align with clinically relevant regions:

• Epidural Hematoma (EDH): The model focuses on the characteristic lens-shaped hyperdense collection between the skull and dura mater, typically appearing as a biconvex collection with well-defined margins.
• Subdural Hematoma (SDH): Attention is directed toward the crescent-shaped hyperdensity along the cerebral convexity, crossing suture lines but respecting dural reflections.
• Subarachnoid Hemorrhage (SAH): The model highlights hyperdense material within the sulci and basal cisterns, corresponding to blood accumulation in the subarachnoid space.
• Intraparenchymal Hemorrhage (IPH): Focus is on hyperdense parenchymal collections within the brain tissue itself, often surrounded by hypodense edema.
• Intraventricular Hemorrhage (IVH): Attention centers on hyperdense material within the ventricular system, particularly casting the occipital horns of the lateral ventricles.
• These visualizations confirm that the ensemble model bases its decisions on radiologically meaningful features rather than spurious correlations or imaging artifacts, supporting the radiological plausibility of the model attention patterns, although clinical validation is still required.

## 5. Discussion

This study developed an explainable multi-model ensemble framework for the six-class classification of non-contrast computed tomography images, including epidural hematoma, subdural hematoma, subarachnoid hemorrhage, intraparenchymal hemorrhage, intraventricular hemorrhage, and normal cases. The proposed ensemble combined InceptionV3, Xception, and InceptionResNetV2 through a soft-voting strategy and achieved an accuracy of 95.34% on the independent test set. The ensemble also obtained precision, recall, and F1-score values of approximately 95%, with a 95% confidence interval of 94.3–96.3%. These findings indicate strong internal test-set performance for multi-class hemorrhage classification.

The performance of the proposed ensemble can be contextualized in relation to previously published deep-learning models for intracranial hemorrhage analysis. Several studies have reported high accuracy for binary hemorrhage detection, with AUC values approaching 0.99 in large-scale clinical datasets [19]. Other investigations have evaluated multi-class classification of hemorrhage subtypes using individual CNN architectures or ensemble strategies. For example, Hussain et al. [12] proposed an attention-based ResNet framework combined with PCA and DCGAN-based data balancing and reported subtype-specific accuracies ranging from 96.1% to 99.2%. Kuo et al. [15] evaluated a cross-architecture system involving EfficientNet-B4, Xception, and InceptionV3 and achieved an accuracy of 94.2% for acute intracranial hemorrhage detection. Hybrid approaches combining CNNs with recurrent components or radiomics features have also been investigated for hemorrhage diagnosis [13,21].

The individual model results provide insight into the behavior of the selected architectures. Xception achieved the highest accuracy among the individual models, at 93.779%, with an AUC of 0.9956. InceptionV3 achieved an accuracy of 92.674% and the highest individual AUC of 0.9961, indicating best discrimination across the six diagnostic categories. InceptionResNetV2 achieved an accuracy of 87.151% and an AUC of 0.9893. Although InceptionResNetV2 showed lower standalone performance, it was retained in the ensemble because ensemble design should consider prediction complementarity in addition to individual accuracy. The three architectures use different feature-extraction mechanisms. InceptionV3 captures multiscale patterns through factorized convolutional branches, Xception extracts fine-grained spatial features using depthwise separable convolutions, and InceptionResNetV2 combines multiscale Inception modules with residual connections.

The ensemble achieved an accuracy of 95.34%, exceeding all individual models. The class-specific AUC values of the ensemble ranged from 0.9933 to 0.9993, demonstrating excellent discriminative ability across all categories. These results are consistent with the hypothesis that combining complementary feature representations can reduce architecture-specific errors and improve classification stability.

The comparison between the individual architectures and the full ensemble can be interpreted as an architecture-level ablation analysis. In this view, each single model represents a degenerate ensemble consisting of only one architecture, whereas the proposed system corresponds to the full configuration with all three components. The ensemble achieved an accuracy of 95.35% (95% CI: 94.3–96.3%), surpassing the accuracies of Xception (93.78%), InceptionV3 (92.67%), and InceptionResNetV2 (87.15%). These results support the hypothesis that different CNN architectures learn complementary feature representations, and that their combination via unweighted soft voting reduces architecture-specific errors and improves classification stability at the slice level.

The class-wise results showed strong performance across most diagnostic categories. The ensemble achieved F1-scores of 97% for EDH, 97% for normal cases, 91% for IPH, 96% for IVH, 97% for SAH, and 95% for SDH. The high performance for normal cases indicates that the model rarely classified non-hemorrhagic scans as hemorrhagic. SAH was also consistently classified with high performance, which may be related to the relatively characteristic appearance of hyperdense blood within the sulci and basal cisterns. The strong results for EDH and IVH further suggest that the ensemble was able to learn the spatial patterns associated with extra-axial and intraventricular hemorrhage.

IPH remained the most challenging category for the individual models. The individual F1-scores for IPH were 89% for Xception, 75% for InceptionResNetV2, and 85% for InceptionV3, whereas the ensemble achieved an F1-score of 91% and an AUC of 0.9933. The lower performance of the individual models may reflect the substantial radiological heterogeneity of IPH. Its appearance on NCCT can vary according to the stage of hemorrhage, anatomical location, lesion size, surrounding edema, and the presence of mass effect. IPH may occur in the basal ganglia, thalamus, brainstem, or lobar parenchyma, producing diverse visual patterns within a single diagnostic category.

The class-specific precision and recall values also revealed complementary error patterns among the individual models. InceptionResNetV2 obtained an IPH precision of 63% and a recall of 93%, suggesting a tendency to classify some non-IPH cases as IPH. Xception and InceptionV3 achieved IPH precisions of 84% and 78%, respectively. In contrast, the ensemble achieved 91% precision and 92% recall for IPH, indicating a more balanced classification profile. This improvement suggests that the voting strategy may have reduced some of the architecture-specific errors. However, the remaining performance gap also indicates that image-only classification may be insufficient for all IPH presentations. Clinical variables such as patient age, symptoms, blood pressure, trauma history, anticoagulant use, and hemorrhage location could provide additional information for future multimodal models.

The ROC analysis further supported the strong discriminative performance of the evaluated architectures. The individual models achieved AUC values of 0.9956 for Xception, 0.9893 for InceptionResNetV2, and 0.9961 for InceptionV3. The ensemble produced class-specific AUC values between 0.9933 and 0.9993. These findings indicate that the models maintained well class separation across a broad range of classification thresholds. The slightly higher AUC of InceptionV3, despite its lower accuracy than Xception, illustrates that AUC and accuracy measure different aspects of performance. Accuracy depends on the selected decision threshold, whereas AUC summarizes discrimination across thresholds. Therefore, both threshold-dependent and threshold-independent metrics should be considered when evaluating multi-class medical-imaging models.

The improvement achieved by the ensemble may be explained by the complementary strengths of its component architectures. Xception can efficiently capture fine spatial details and lesion boundaries through depthwise separable convolutions. InceptionV3 can represent localized and more widespread abnormalities through multiscale factorized convolutional branches. InceptionResNetV2 combines multiscale processing with residual pathways, which may provide additional hierarchical representations.

The Grad-CAM analysis provided qualitative evidence that the ensemble focused on anatomically plausible regions associated with the predicted hemorrhage classes. For EDH, the attention was generally directed toward the characteristic biconvex extra-axial collection. For SDH, the model focused on the crescent-shaped collection along the cerebral convexity. For SAH, the attention was concentrated in the sulci and basal cisterns, whereas for IPH it was directed toward hyperdense parenchymal regions. For IVH, the model focused on the ventricular system. These patterns are consistent with the expected radiological distribution of the respective hemorrhage types.

However, Grad-CAM should be interpreted as a qualitative localization method rather than definitive evidence of causal reasoning or clinical validity. Although the attention maps appeared radiologically plausible, they do not prove that the model is free from dataset-specific confounding factors such as scanner-related patterns, image artifacts, skull characteristics, or acquisition-related differences. Quantitative localization analysis, independent radiologist assessment, calibration analysis, and prospective reader studies would be required to determine whether these explanations reliably support clinical decision-making.

The findings have potential relevance for computer-aided analysis of emergency head CT examinations. Rapid identification of hemorrhage and preliminary classification of its subtype may assist radiologists in prioritizing examinations and reviewing subtle or ambiguous findings. However, the current framework should be regarded as a research-stage decision-support system rather than an autonomous diagnostic tool. The model was trained and evaluated using 2D NCCT slices from a single public dataset, and it did not incorporate the three-dimensional volumetric context used in routine radiological interpretation. In addition, the dataset did not represent the full diversity of institutions, scanners, patient populations, imaging protocols, and disease prevalences encountered in clinical practice.

An important methodological consideration is that the proposed framework operates on individual 2D NCCT slices, whereas hemorrhage diagnosis in clinical practice is inherently volumetric and based on the joint interpretation of multiple consecutive slices within a CT examination. The public dataset used in this study was provided as de-identified 2D images without DICOM headers, slice thickness, slice spacing, slice order, or identifiers linking slices to the same patient or examination. As a result, it was not possible to reconstruct 3D volumes, to model inter-slice dependencies, or to aggregate slice-level predictions into examination-level diagnoses.

The decision to adopt a 2D slice-based formulation was therefore driven by the structure and metadata limitations of the available dataset, as well as by practical considerations. Many previous deep-learning studies on head CT hemorrhage have similarly used 2D slice-based classification when working with publicly available, de-identified image repositories, and have demonstrated that 2D CNNs can learn discriminative features for hemorrhage detection and subtype classification. Nevertheless, we acknowledge that the lack of volumetric modeling and examination-level aggregation is an important limitation that may affect the clinical interpretability and generalizability of the reported performance.

The proposed ensemble framework aligns with recent methodological trends in neuroimaging deep learning. Studies on brain-tumor MRI classification have shown that multi-architecture ensembles and attention mechanisms can improve both accuracy and interpretability relative to single models [23,24,25]. Similarly, recent intracranial-hemorrhage CT studies have emphasized the value of external validation, patient-level evaluation, and quantitative explainability metrics beyond qualitative heatmaps [26,27]. Compared with these works, our study contributes a six-class ensemble with class-wise AUC and Grad-CAM visualization, but shares the limitation of relying on a single public dataset without external validation.

To facilitate direct comparison with prior work on the same publicly available dataset, we summarize in Table 7 the performance of our proposed ensemble model alongside previously reported results that used the Brain Hemorrhage Multiclass dataset. Because the number of studies reporting multi-class results on this exact dataset is limited and because preprocessing, partitioning, and evaluation protocols may differ, the comparison should be interpreted as indicative rather than definitive.

## 6. Limitations

Several limitations should therefore be considered. First, the study used a single publicly available dataset, and no external validation was performed. Second, the analysis was conducted at the image-slice level rather than at the patient or complete examination level. The dataset did not contain PatientID, ExaminationID, accession number, study identifier, or another reliable grouping variable. Therefore, we could not perform a group-wise split before image sampling or verify that slices from the same examination were restricted to one subset. Similar or adjacent slices from the same examination may consequently have been distributed across the training, validation, and test sets. This possibility introduces a risk of inter-subset dependence and may have resulted in optimistic estimates of model performance. The reported metrics should therefore be interpreted as internal image-level results and not as estimates of patient-level clinical generalization. Third, all reported results are based on a single training run per architecture with a fixed random seed and a single train/validation/test partition. Repeated experiments with multiple random seeds were not performed due to the computational cost of training three deep architectures on 34,366 images. Consequently, the reported confidence intervals reflect uncertainty due to test-set sampling under a fixed partition, but do not capture variability arising from different data splits or initialization seeds. This limitation should be considered when interpreting the modest performance differences between the ensemble and the best individual model. Fourth, the study did not include a formal ablation analysis to quantify the specific contribution of each architecture to the ensemble. Finally, Grad-CAM provides coarse localization and does not constitute a complete explanation of the model’s decision process. Although Grad-CAM heatmaps appeared radiologically plausible in selected cases, they should not be interpreted as definitive evidence of causal reasoning or clinical validity without quantitative localization metrics and reader studies.

## 7. Future Works

Future research should focus on multi-institutional and prospective validation using datasets that provide reliable patient-level and examination-level identifiers together with complete DICOM metadata. In such datasets, grouping should be performed before image sampling, and all slices from the same patient or examination should be assigned exclusively to the training, validation, or test set. This would enable the reconstruction of full CT volumes and the evaluation of 3D CNNs or hybrid 2D–sequence models.

In addition, future studies should investigate aggregation strategies that combine slice-level predictions into examination-level diagnoses, for example through majority voting, maximum probability, or attention-based fusion across consecutive slices. Such approaches would provide a more realistic estimate of clinical performance and better align the evaluation protocol with routine radiological practice. Incorporating volumetric information, clinical metadata, and multimodal features may further improve performance for heterogeneous categories such as IPH. also future studies should repeated experiments with multiple random seeds and apply formal statistical tests (e.g., paired bootstrap or McNemar’s test) to assess whether the improvement of ensemble methods over individual architectures is statistically significant and robust across different data partitions.

## Figures and Tables

**Table 1:** Comparison of representative previous studies.

Study	Dataset/Task	Classes	Main Method	Reported Performance	Explainability	Main Limitation
Grewal et al. [7]	Head CT hemorrhage detection	Binary	RADnet; recurrent attention-based DenseNet	Radiologist-level detection performance	Not systematically evaluated	Primarily focused on detection rather than complete subtype classification
Ammar et al. [8]	CT-based ICH classification	Multiple CNN architectures	Comparative evaluation of CNN models	Best accuracy of 96% using VGG16	Not reported	Comparison across architectures was performed on a specific dataset and protocol
Akram et al. [9]	Acute hemorrhage detection and subtype classification	Multiple hemorrhage types	Double-branch Xception architecture	High subtype-classification performance	Not reported	Complex architecture and limited evidence of external generalizability
Hassan and Aljobouri [11]	CT-based hemorrhage classification	Selected subtypes	InceptionResNetV2 and DenseNet201	High performance across evaluated classes	Not reported	Did not evaluate all major hemorrhage types and normal cases together
Hussain et al. [12]	Acute hemorrhage detection and classification	Multiple hemorrhage types	Attention-based ResNet, PCA, and DCGAN	Subtype-specific accuracies of 96.1–99.2%	Limited	Interpretability and external validation were limited
Jena et al. [13]	CT-based ICH diagnosis	Hemorrhage-related classes	Transfer learning and hybrid CNN–Bi-LSTM/radiomics	Approximately 93% accuracy	Not clearly reported	Hybrid design and task definition may limit direct comparison
Gautam and Raman [14]	Hemorrhagic and ischemic stroke classification	Binary/multiclass stroke categories	CNN with image-fusion preprocessing	Competitive classification performance	Not reported	Focused on broader stroke classification rather than six-class ICH classification
Kuo et al. [15]	Acute ICH detection on head CT	Primarily detection-oriented	EfficientNet-B4, Xception, and InceptionV3	94.2% accuracy in the reported setting	Not emphasized	Primarily detection-oriented and not focused on comprehensive subtype classification
Arbabshirani et al. [19]	Head CT ICH detection	Binary	Deep learning integrated into clinical workflow	AUC of approximately 0.99	Not reported	Focused mainly on binary detection
Thabarsa et al. [21]	Etiological classification of spontaneous ICH	Multiple etiologies	Radiomics and clinical metadata	79% accuracy	Feature-based interpretation	Not an end-to-end deep-learning image-classification framework

**Table 3:** Evaluation Metrics (All metrics are computed in a one-vs-rest manner for the six-class problem and then macro-averaged unless otherwise stated).

Metric	Formula
Accuracy	(TP + TN)/(TP + TN + FP + FN)
Precision	TP/(TP + FP)
Recall (Sensitivity)	TP/(TP + FN)
Specificity	TN/(TN + FP)
F1-score	2 × (Precision × Recall)/(Precision + Recall)

TP = True Positive. TN = True Negative. FP = False Positive. FN = False Negative.

**Table 4:** Performance metrics of individual models on the NCCT test set.

Model	Accuracy (%)	Precision (%)	Recall (%)	F-Score (%)	CI 95%	AUC
Xception	93.779	94	94	94	[92.6%, 94.9%]	0.9956
InceptionResNetV2	87.151	90	87	87	[85.5%, 88.7%]	0.9893
InceptionV3	92.674	93	93	93	[91.4%, 93.9%]	0.9961
Ensemble	95.348	95	95	95	[94.3%, 96.3%]	0.9970

**Table 5:** Class-wise performance (one-vs-rest) of the CT model.

Model	Class	Precision (%)	Recall (%)	F1-Score (%)	AUC
Xception	EDH	96	97	97	0.9991
	Healthy	97	96	96	0.9983
	IPH	84	95	89	0.9904
	IVH	93	94	94	0.9959
	SAH	98	93	95	0.9972
	SDH	96	88	92	0.9924
InceptionResNet-V2	EDH	95	82	88	0.9919
	Healthy	99	99	99	0.9998
	IPH	63	93	75	0.9706
	IVH	94	87	90	0.9944
	SAH	94	93	94	0.9942
	SDH	97	67	79	0.9848
InceptionV3	EDH	96	84	90	0.9966
	Healthy	98	98	98	0.9997
	IPH	78	93	85	0.9892
	IVH	94	97	95	0.9981
	SAH	98	94	96	0.9981
	SDH	97	88	92	0.9948
Ensemble	EDH	96	97	97	0.9993
	Healthy	96	98	97	0.9991
	IPH	91	92	91	0.9933
	IVH	95	97	96	0.9982
	SAH	99	95	97	0.9986
	SDH	96	93	95	0.9934

**Table 6:** Class-wise performance (one-vs-rest) of the ensemble model.

Model	Class	Precision (%)	Recall (%)	F1-Score (%)	AUC
Ensemble	EDH	96	97	97	0.9993
	Healthy	96	98	97	0.9991
	IPH	91	92	91	0.9933
	IVH	95	97	96	0.9982
	SAH	99	95	97	0.9986
	SDH	96	93	95	0.9934

**Table 7:** Comparison of studies using the same Brain Hemorrhage Multiclass dataset.

Study	Classes	Main Method	Accuracy (%)	Explainability	Note
Ammar et al. [8]	5–6 (multi-class ICH)	Comparative CNNs (VGG16 best)	~96	Not reported	Used a subset of the dataset; exact partitioning not fully specified
Akram et al. [9]	Multiple hemorrhage types	Double-branch Xception	>98 (subtype-specific)	Not reported	Focused on selected subtypes; evaluation protocol differs
Hassan & Aljobouri [11]	Selected subtypes	InceptionResNetV2 + DenseNet201	>96	Not reported	Did not include all six classes simultaneously
Proposed ensemble	6 (EDH, SDH, SAH, IPH, IVH, Normal)	InceptionV3 + Xception + InceptionResNetV2 (soft voting)	95.35	Grad-CAM	Internal test set; no external validation

## Data Availability

The Brain Hemorrhage Multiclass dataset used in this study is publicly available through Kaggle at https://www.kaggle.com/datasets/yogeshkn/brain-hemorrhage-multiclass (Version 1, released on 31 March 2025; accessed on 3 August 2026) and is distributed under the Apache 2.0 license. The dataset contains de-identified brain CT images and was used for secondary analysis following ethical approval by Kerman university of medical science, Approval Number IR.KMU.REC.1404.464. The annotations were generated or validated by expert radiologist. No additional non-public datasets were used. The source code of the current study is available on GitHub. All experiments were implemented in Python 3.12 using TensorFlow 2.19.0 with CUDA. The hardware configuration consisted of an Tesla A100 GPU with 32 GB of memory, 12 GB of VRAM.
